# Home Automation for Adults With Disability Following an Injury: Protocol for a Social Return on Investment Study

**DOI:** 10.2196/42493

**Published:** 2022-12-21

**Authors:** Jenny Cleland, Claire Hutchinson, Patricia A H Williams, Kisani Manuel, Kate Laver

**Affiliations:** 1 College of Medicine and Public Health Flinders University Adelaide Australia; 2 Caring Futures Institute College of Nursing and Health Sciences Flinders University Adelaide Australia; 3 College of Science and Engineering Flinders University Adelaide Australia; 4 Department of Rehabilitation, Aged Care and Palliative Care Flinders Medical Centre Adelaide Australia

**Keywords:** disability, serious injury, economic evaluation, home automation, long-term care, social return on investment, injury, technology, community, Australia, decision-making

## Abstract

**Background:**

People with disability following a serious injury require long-term care. The most common injuries resulting in long-term disability are spinal cord and acquired brain injuries. While the long-term effects are difficult to predict and will vary between individuals, the costs of care and recovery span well beyond the initial treatment phase and include long-term care. Long-term care is changing with the availability and advances in cost and function of technologies, such as home automation. “Home automation” refers to technology that automates or remotely controls household functions. Home automation costs vastly differ, but home automation has the potential to positively impact the lives of people with disabilities. However, there is a dearth of evidence relating to the impact of home automation for people with a disability and few rigorous evaluations about the costs and return on investment.

**Objective:**

The purpose of this study is to describe the impact of home automation for people with long-term disability following a serious injury (such as a motor vehicle accident) using case studies, and by conducting an evaluation of the costs and outcomes for individuals, families, and the wider community using a Social Return on Investment (SROI) approach.

**Methods:**

SROI is a form of economic evaluation that develops a theory of change to examine the relationship among inputs, outputs, and outcomes and, in recent years, has gained popularity internationally, including in Australia. SROI has six phases: (1) identify scope and stakeholders, (2) map outcomes, (3) evidence outcomes and give them value, (4) establish impact, (5) calculate the SROI, and (6) report findings. Individuals with a disability who use home automation and key stakeholders will be interviewed. Stakeholders will be individuals involved in home automation for people with disabilities, such as allied health professionals, medical practitioners, equipment suppliers, engineers, and maintenance professionals. Users of home automation will be people who have a disability following a serious injury, have the capacity to provide consent, and have 1 or more elements of home automation. The impact of home automation will be established with financial proxies and appropriate discounts applied to avoid overestimating the social return. The SROI ratio will be calculated, and findings will be reported.

**Results:**

The project was funded in November 2021 by the Lifetime Support Authority. Recruitment is underway, and data collection is expected to be completed by October 2022. The final results of the study will be published in March 2023.

**Conclusions:**

To our knowledge, this study represents the first study in Australia and internationally to employ SROI to estimate the social, personal, and community outcomes of home automation for people with a disability following a serious injury. This research will provide valuable information for funders, consumers, researchers, and the public to guide and inform future decision-making.

**International Registered Report Identifier (IRRID):**

DERR1-10.2196/42493

## Introduction

People with a disability following a serious injury (such as a motor vehicle accident) require treatment, care, and support. The most common injuries resulting from an accident are spinal cord injuries and acquired brain injuries [1]. While the long-term effects are difficult to predict and vary between individuals, the costs of care and recovery span well beyond the initial treatment phase and include long-term care [2,3]. For many years after an injury, people with disabilities may require assistive equipment, home modifications, and attendant care, as well as informal care provided by family members or friends [4]. Long-term care for people with disabilities can be costly. For example, data from the United Kingdom suggest the most significant cost of long-term care for people with acquired brain injury is the cost of care attendants, which comprises approximately 80% of costs [3]. However, long-term care for people with disabilities following a serious injury is changing with the availability of new technologies.

“Home automation” refers to technology that automates or remotely controls household functions [5,6]. For example, controlling doors, blinds, heating and cooling, lighting, windows, doorbells, intercom systems, taps, and entertainment systems. The advances in both the cost and functionality of technology have made home automation more accessible. Acquiring home automation products requires several stages, such as assessment and selection, authorization and acquisition, implementation and training, and review and maintenance. It involves multiple people, such as the home automation user, family or support network, funder, allied health professional, medical practitioner, equipment supplier, and installer [7].

Home automation costs vastly differ, and the potential benefits of home automation can be wide ranging. These may include improved safety, improved comfort, greater independence and autonomy, improved social participation, improved quality of life, and the potential for the person with disability to be left alone for longer periods of time, leading to a reduced need for care attendants [6-13]. For example, in adopting a modeling approach to examine the trade-off between home automation and informal and formal care, Agree and colleagues [8] found that home automation use was associated with reduced hours of personal care for people with disabilities, particularly for those with high education levels, were unmarried, and had good cognition. In a study evaluating the impact of home automation for people with spinal cord injuries, functional abilities to perform daily tasks were improved, and home automation was identified as positively impacting individuals’ psychosocial health, perceptions of quality of life, and independence [12]. Indeed, a recent literature review of the relationship between home automation and disability provided evidence to support a strong relationship between home automation and increased independence, leading to improved social inclusion of people with disabilities [6]. However, despite the many benefits, there appear to be few users of home automation, and electronic assistive technologies remain underused in long-term care settings [6,7].

There have been an increasing number of studies evaluating smart homes and home automation in different populations. A recent scoping review examining the effectiveness of smart home technologies for older people with dementia identified 5 studies that provided evidence to support the use of smart home technologies in improving their health outcomes [14]. A qualitative study by Dermody and colleagues [15] explored the uptake and perceptions of home automation for older adults living in the community. The findings suggested that older people were receptive to using home automation to increase their independence, despite some minor concerns about their privacy and personal safety. However, increased information and support were required in order to adopt home automation successfully [15]. A recent scoping review on the impact of smart home and communication technology devices and systems for people with disabilities identified 21 studies. Notably, of the 21 studies, only 2 specifically focused on the impact of home automation technology for people with disabilities, and neither included a cost nor a return on investment component [16]. Overall, there is a gap in the current research literature on rigorous evaluations and information about the costs and return on investment [16,17].

In Australia, funding for home automation for people with disabilities is provided by the National Disability Insurance Agency (NDIA), an Australian government organization that funds costs associated with disability, with individual state organizations such as the Lifetime Support Authority (LSA) in South Australia funding home automation for people who sustain serious injuries following a motor vehicle accident. Internationally, funding for home automation is available in high-income countries. For example, local government councils provide grants for home automation in the United Kingdom, and support is also available through registered disability charities (National Health Service [18]). Similarly, in the United States, home automation is funded by individual state programs for assistive technology [19]. Home automation has great potential to positively impact the lives of people with disabilities.

The overarching aim of the study is to understand the impact of home automation on people with disabilities following a serious injury. The objectives of the study are as follows: (1) to describe the impact of home automation for people with long-term disability following a serious injury (eg, acquired through a motor vehicle accident) using case studies, and (2) to evaluate the Social Return on Investment (SROI) of home automation for this population. The findings from this study will provide valuable and significant information for funders, consumers, researchers, and the public to guide future decision making about home automation.

## Methods

### Design and Methodology

A steering group will be established at project commencement, involving the research project team, representatives from the funding body (LSA), a disability advocacy group, and an equipment supplier. The steering group will also include 2 consumer representatives who have experienced serious injury or care for someone with serious injury. The steering group will oversee the conduct of the study, provide accountability and transparency, inform the analysis, and support the dissemination of findings.

The study will employ a SROI approach. The SROI methodology was developed in the United States in 2000 by the Roberts Enterprise Fund [20] and subsequently refined and tested in the United Kingdom by the New Economics Foundation [21]. The approach is widely implemented in the United Kingdom due to the Department of Health encouraging health and social care research to adopt the SROI methodology and the creation of the Social Enterprise Investment Fund, which provides funding and support to organizations adopting this approach [20,22]. In recent years, SROI has gained popularity internationally, including in Australia [23-25].

SROI is a form of evaluation that commences with a theory of change to examine the relationship among inputs, outputs (activities), and outcomes. The approach is unique as it emphasizes the engagement of stakeholders to inform the analysis and for the valuation of personal, community, and societal outcomes that are not typically included in more traditional forms of economic evaluation (for example, cost-utility analysis, cost-benefit analysis, and cost-effectiveness analysis). The method captures the overall social impact of an intervention in a simple ratio such as 4:1 (indicating that Aus $4 [US $2.68] of social value has been created for every Aus $1 [US $0.67] invested). The SROI methodology has six distinct phases: (1) identify scope and stakeholders, (2) map outcomes, (3) evidence outcomes and give them value, (4) establish impact, (5) calculate the SROI, and (6) report findings [26].

#### Stage 1—Identify Scope and Stakeholders

Key stakeholders in home automation will be identified and invited to participate in interviews (n=10) to determine inputs (eg, assessment procedures, costs of home automation, installation and ongoing costs, time involved in the process), and intended outcomes for the home automation user. Stakeholders will be individuals involved in home automation (designer, prescriber, advisor, and installer) for people with serious disabilities, such as allied health professionals, medical practitioners, equipment suppliers, rehabilitation engineers, installers, and service and maintenance professionals.

Users of home automation will be interviewed (n=5) to determine the outcomes of home automation. The selected sample size considers the relatively small population size of people with disabilities following a serious injury requiring high levels of care and the small subgroup that uses home automation [7]. Participants will be people who have a disability following a serious injury, have the capacity to provide consent to participate in the research, and have 1 or more elements of home automation. Where appropriate, interviews will be conducted with family members. This will be deemed appropriate when the person with disability provides consent for the family member to participate in the interview and when the family member has a good understanding of the person’s home automation and daily activities.

Interviews will be semi-structured, and the interviewer will follow an interview schedule. The interviews will be conducted face to face, over the phone, or via videoconference (depending on the participant’s location and preference) at a time convenient to the participant. All interviews will be audio recorded and professionally transcribed. The interview schedule will consist of 4 sections: socio-demographic questions, inputs of home automation, outputs (activities) of home automation, and the outcomes experienced (capturing negative as well as positive outcomes). The participant will also have the opportunity at the end of the interview to discuss any impacts and outcomes of home automation that have not already been discussed.

Purposive sampling will be adopted to recruit stakeholders and home automation users for the interviews. Recruitment for users of home automation will initially be done via LSA. In addition, local providers of home automation and disability organizations will be contacted to inform them about the research and to advertise the study to potential participants by sharing information, using the opt-in approach. Finally, social media (Facebook and Twitter advertisements for consumer groups) will be used to promote the research and recruit participants. Stakeholders will be recruited through the NDIA, professional networks, the LSA, organizations that provide home automation, and the steering group. An email will be sent to potential participants with information about the study, allowing them to opt in by replying to the research team. If necessary, social media (Facebook and Twitter) will be used to promote the research and advertise for potential stakeholder participants.

#### Stage 2—Map Outcomes

The inputs, outputs (activities), and outcomes involved in the home automation process will be mapped (this is referred to as the “theory of change”), guided by the interview data from stage 1, existing literature, and with input from the steering group. Given the wide variety of home automation options, 4 scenarios will be developed based on LSA’s most common injury types relevant to requiring home automation (spinal cord injury and acquired brain injury) and the differing levels of severity of the injury and the type of home automation. The data from the existing literature and interviews will be used to develop the scenarios, and the inputs, outputs, and outcomes will be described in relation to these scenarios to provide context for the analysis and to support the interpretation of findings. Tables will be produced to illustrate the funder’s inputs (costs to the funder and costs of professional services to support the use of home automation), the calculations for each scenario, and the payback period for each scenario. A scenario-based approach was recently used successfully in a SROI analysis calculating the social impact of modified vehicles for people with disabilities, in which 5 scenarios were developed to illustrate the social return for low to high-cost modifications of vehicles [23]. A significant advantage of adopting a scenario-based approach in SROI analysis is that findings are more generalizable [23,26].

#### Stage 3—Evidence Outcomes and Give Them Value

Evidence of outcomes will be obtained from stage 1 interview data as well as relevant Australian and international data from published home automation studies. Values for outcomes will be obtained in two ways: (1) from extensive searches of existing SROI literature reporting the same or similar outcomes and how they were valued, and (2) from value games with participants and their families. Value games are a revealed preference approach in which participants rank an outcome without considering its market value among several items that can be purchased [27,28]. In this way, the unique value of outcomes for this population can be identified.

#### Stage 4—Establish Impact

The impact of home automation will be established with appropriate discounts applied to cumulated financial proxies to avoid possible overclaiming. Discounts are calculated based on what would have happened without the intervention (deadweight), what outcomes were displaced by the intervention (displacement), who else has contributed to the outcomes aside from the funder (attribution), and whether the experience of the outcomes declines over time (drop off). The “benefit period” (the period over which the social return will be calculated) will be determined. Benefit periods need to be long enough to capture the most valuable outcomes for participants, and in this context, consider the life span of home automation products.

#### Stage 5—Calculate the SROI

The SROI ratio is calculated with costs incurred and benefits realized at different time periods made comparable using discounting to calculate the net present value to ensure the values of the outcomes are in today’s dollars. Sensitivity analysis will also be performed to determine how sensitive the SROI ratio is to changed assumptions in the calculation [27,29,30]. The payback period will also be established for each scenario to understand the period over which the costs of the intervention are “paid back” in accumulated social value.

#### Stage 6—Report Findings

The results will be widely disseminated in different formats for different audiences. Findings will be presented in project reports and in peer-reviewed articles. Findings will also be disseminated at relevant conferences. It is also anticipated that the Flinders University media team will share the findings internally and externally to promote the research.

### Ethical Considerations

Ethical approval (human subject research) was obtained by the Flinders University Social Human Research Ethics Committee in South Australia (project number 5039) in March 2022. Participants will give informed consent and can opt out of the research. Participant’s information will remain private and any identifying details will be omitted. It is acknowledged that this research is of a sensitive nature, and participants may become upset or distressed during the interview. Participants who become upset will be comforted by the interviewer, offered details on counseling services, and may withdraw from the research if they wish. The study will include people with significant disabilities, and therefore, the interviews will be accommodating to ensure the comfort of the participant, for example, by monitoring fatigue and shorter interviews. It is also the intention that interviews will be conducted with family members on behalf of the person with the disability if this is a preferred option. Participants will also be given the option of requesting another person to be present at the interview if they wish. Potential participants will be given a participant information sheet and a consent form. All participants will be provided with the researcher’s contact information and an opportunity to ask questions and discuss any aspect of the study. All study data will be stored confidentially, and any identifying data will be removed to ensure the data is deidentified. Any publications arising from the data will not identify any individual person. Informed written consent to participate in the interviews and for the use of this data for analysis will be sought from all participants. In recognition of the participant’s time and contribution, they will receive an honorarium (Aus $50 [US $35] voucher) on completion of the interview.

## Results

The project was funded in November 2021 by LSA (Grant No. R21005). Recruitment of participants for the interviews commenced in June 2022, and we expect data collection to be completed by October 2022. As of September 2022, we have recruited 10 stakeholders and 6 users of home automation. Data analysis will be conducted during November and December 2022, with the results expected to be published in March 2023.

## Discussion

The aim of the study is to understand the impact of home automation for people with disabilities following a serious injury, and this will be achieved by describing the impact using case studies and evaluating the SROI of home automation for this population.

To our knowledge, this study represents the first study in Australia and internationally to employ a SROI methodology to estimate the social, personal, and community outcomes of home automation for people with a disability following a serious injury. SROI is advantageous because it involves both consumers and stakeholders in the analysis and valuation of personal, community, and societal outcomes, resulting in a simple ratio that demonstrates how much social value (in Aus $) is created for every Aus $1 [US $0.67] invested.

Home automation is tailored depending on an individual’s needs and can range from inexpensive (Aus $200 [US $130]) to more costly (Aus $25,000 [US $20,000]) depending on the type of home automation [7]. Given the wide range of types of home automation and variations in costs, the SROI analysis will adopt a scenario approach that represents low-cost home automation through to high-cost home automation. A benefit of this approach is that the findings will be more generalizable as the SROI analysis is not based on a single home automation scenario [23].

Home automation has the potential to offer significant benefits to people with disabilities. The World Health Organization (WHO) states home automation enables people with a disability to experience increased independence, improved health, and well-being and can also lead to a reduction in caregiver hours, impacting the broader community [13]. Recognizing the challenges encountered by individuals when accessing assistive technology in some countries, the WHO has introduced the Global Cooperation on Assistive Technology. This initiative aims to provide improved access to assistive technology for people with disabilities worldwide by reinforcing the WHO’s existing strategies on people-centered and integrated health services and disability [13]. In Australia, agencies such as the NDIA advocate for access to assistive technology for people with disabilities to improve their economic and community participation, benefiting not only the individual but also their family and the wider community.

Upon completion of the study, the strengths and limitations will be summarized. The research builds on what is already known about home automation by considering the benefits of home automation reported by users in the research literature. Results will provide valuable information for funders, consumers, researchers, and the public to guide and inform future decision-making and practice about home automation. By providing a simple social return ratio, the payback period can be calculated, and comparisons between high-cost and low-cost home automation can be made to guide decisions. Furthermore, the study may be helpful to researchers who want to assess the social value of other types of interventions, and the model could be adapted for research in other fields.

## Abbreviations

LSA: Lifetime Support Authority
NDIA: National Disability Insurance Agency
SROI: Social Return on Investment
WHO: World Health Organization

## Data Availability

Data sharing is not applicable to this article as no data sets were generated or analyzed during the current study.
